# Transformation of ACC into aragonite and the origin of the nanogranular structure of nacre

**DOI:** 10.1038/s41598-017-12673-0

**Published:** 2017-10-05

**Authors:** Elena Macías-Sánchez, Marc G. Willinger, Carlos M. Pina, Antonio G. Checa

**Affiliations:** 1 Department of Stratigraphy and Palaeontology, University of Granada, Granada, 18071 Spain; 2 Andalusian Earth Sciences Institute (IACT), UGR – CSIC, Avd. de las Palmeras 4, Armilla, 18100 Granada, Spain; 3 Department of Inorganic Chemistry, Fritz Haber Institute of the Max Planck Society, Berlin, 14195 Germany; 4 Department of Colloid Chemistry, Max Planck Institute of Colloids and Interfaces, 14476 Potsdam, Germany; 5 Department of Crystallography and Mineralogy, Complutense University of Madrid, Geosciences Institute (IGEO) (UCM-CSIC), E-28040 Madrid, Spain

## Abstract

Currently a basic tenet in biomineralization is that biominerals grow by accretion of amorphous particles, which are later transformed into the corresponding mineral phase. The globular nanostructure of most biominerals is taken as evidence of this. Nevertheless, little is known as to how the amorphous-to-crystalline transformation takes place. To gain insight into this process, we have made a high-resolution study (by means of transmission electron microscopy and other associated techniques) of immature tablets of nacre of the gastropod *Phorcus turbinatus*, where the proportion of amorphous calcium carbonate is high. Tablets displayed a characteristic nanoglobular structure, with the nanoglobules consisting of an aragonite core surrounded by amorphous calcium carbonate together with organic macromolecules. The changes in composition from the amorphous to the crystalline phase indicate that there was a higher content of organic molecules within the former phase. Within single tablets, the crystalline cores were largely co-oriented. According to their outlines, the internal transformation front of the tablets took on a complex digitiform shape, with the individual fingers constituting the crystalline cores of nanogranules. We propose that the final nanogranular structure observed is produced during the transformation of ACC into aragonite.

## Introduction

The production of amorphous minerals by organisms is a long-known fact, but it was not until the pioneering work of Towe and Lowenstam^1^ that evidence for the formation of biominerals by both vertebrates and invertebrates through the corresponding amorphous transient phases began to accumulate.

Calcium carbonate is, with few exceptions, the most common material used by invertebrates to construct their hard structures (shells, plates, spicules, etc.). These are made basically of calcite, aragonite and, very rarely, of vaterite. The first amorphous phase in calcium carbonate systems was detected in the larval spicule of the sea urchin^2^, as electrodense granules inside spiculogenic cell vesicles, which are subsequently transported to the mineralization site^3^. Subsequently, amorphous calcium carbonate (ACC) appears in regenerating adult spines and has been shown to transform into calcite^4^.

Transformation of ACC into aragonite was proposed in embryos of a pulmonate snail^5,6^, in larval bivalves^7^, and in freshwater cultured pearls^8^, based on a large variety of techniques (high-resolution X-ray diffraction, Raman spectroscopy, Scanning and Transmission Electron Microscopy –SEM and TEM)^9^. Although on calcitic materials, the TEM recognition by Baronnet *et al*.^10^ of an amorphous cortex (several tens of nm thick) in the prisms of the pearl oyster *Pinctada margaritifera* is interesting, since the cortex was located exactly at the growth fronts of the prisms. Despite being the most intensively studied natural organic-inorganic biocomposite in invertebrates, the evidence of ACC associated to nacre is scant. Besides the previously mentioned report of ACC granules in pearls by Jacob *et al*.^8^, Nassif *et al*.^11^ found an amorphous rim 3–5 nm thick around mature nacre tablets; they recrystallized the amorphous rim under the electron beam and indexed the recrystallized areas as aragonite. Subsequently, Zhang and Xu^12^ provided images of nacre crystalline nanodomains immersed in amorphous material. Recognition of ACC in nacre was made by DeVol *et al*.^13^. Their photoemission electron spectromicroscopy (PEEM) maps clearly showed that ACC is more abundant in younger tablets (towards the tops of gastropod nacre towers). This is consistent with the fact that ACC transforms progressively into aragonite as tablets age.

There is presently evidence that the formation of biominerals does not seem to proceed via the aggregation of monomers (as postulated by the classical crystallization theory), but by non-classical crystallization via an aggregation-based growth mechanism of precursor nanoparticles. At the same time, the biominerals are characterized by a nanostructure made of tightly-packed nanosized crystalline granules separated by intergranular sheaths of a different nature (usually implied to be organic). This nanotextural imprint is taken as evidence of the particle aggregation mechanism (see the recent comprehensive reviews in^14,15^). In nacre, the nanogranular substructure was revealed time ago^16,17^. Hovden *et al*.^18^ observed that nacre in the bivalve *Pinna nobilis* initiates with nanofibrillar aggregations of nanoparticles, which grade into irregular early-nacre lamellae, and then into well-ordered mature nacre. They interpreted this as evidence that the process is driven by aggregation of nanoparticles (50–80 nm).

Until now, a study combining high-resolution imaging with unambiguous recognition of ACC and its distribution within nacre platelets has been lacking. Following DeVol *et al*.^13^, we have selected incipient nacre tablets, where ACC is more abundant. We have used the relatively common littoral gastropod *Phorcus turbinatus*. By combining different high-resolution Scanning/Transmission Electron Microscopy (S/TEM) techniques, Atomic Force Microscopy (AFM), and elemental analysis, we have visualized for the first time the distribution of the amorphous and crystalline phases at the nanoscale, which has in turn allowed us to elucidate the progressive crystallization mechanism in nacre and relate it with the nanogranular structure of nacre.

## Results

**Figure 1:**
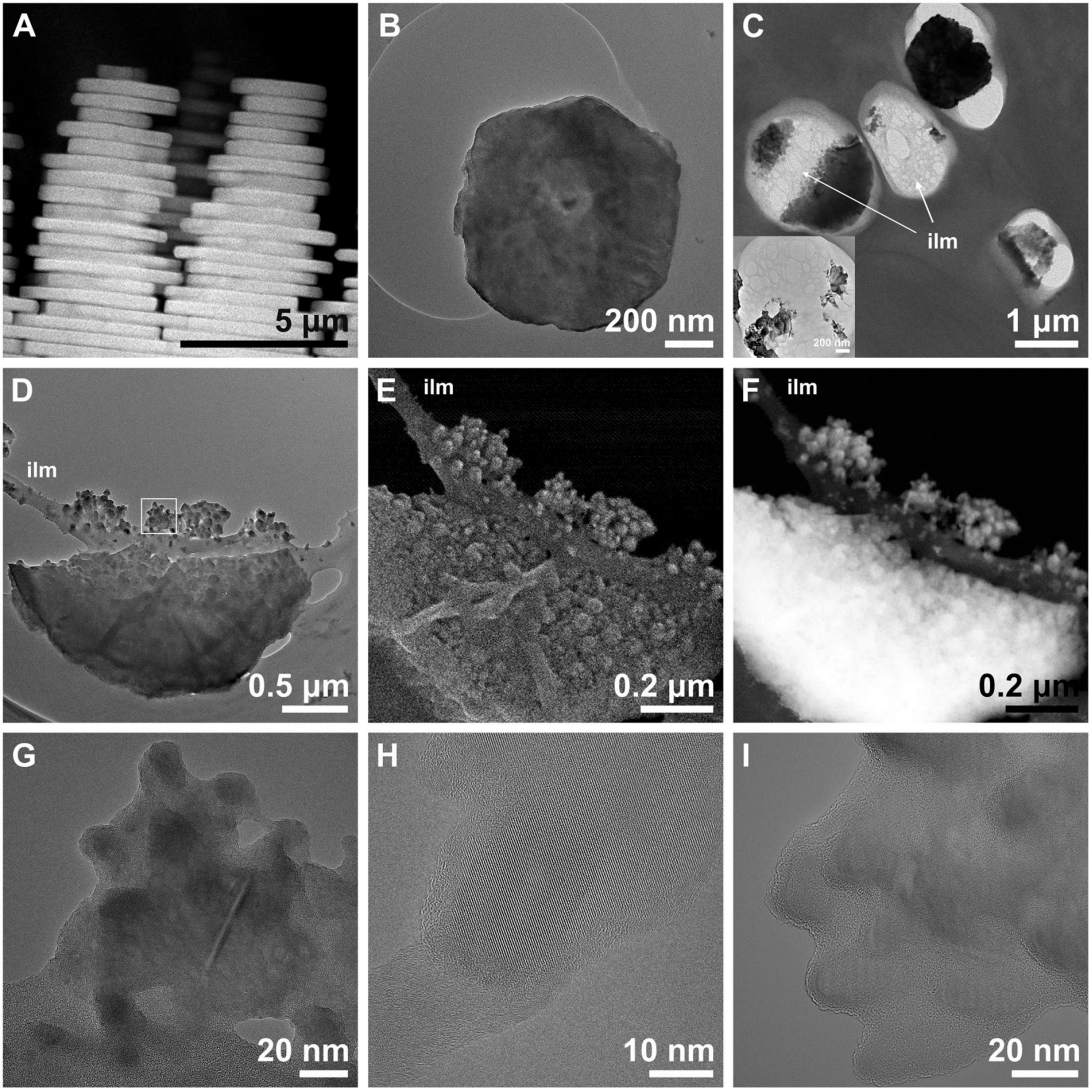
Gastropod nacre tablets from macro- to nanoscale. (**A**) Lateral view of nacre towers (HAADF). (**B**) Tablet at the top of a tower cut almost perpendicular to the growth axis of the tower, with its characteristic pseudohexagonal shape. (**C**) Slightly oblique section of some tablets. The characteristic porous structure of the interlamellar membranes (ilm) is visible. Inset, detail of the porous structure. (**D**,**E**,**F**) Image contrast comparison of a single tablet in TEM (**D**), SE (**E**) and HAADF (**F**). The tablet is composed of a myriad of nanogranules. Note the three-dimensional appearance provided by the SE image (**E**) compared to the other two modes. (**G**) HRTEM of the area framed in (**D**): the nanogranules are composed by crystalline domains embedded in amorphous material. (**H**) Oval shaped crystalline nanodomain showing lattice fringes, with amorphous rims. (**I**) Detail of several crystalline domains embedded in amorphous matter.

Gastropod nacre tablets are stacked in towers^19^ (Fig. 1A), each tablet having a pseudo-hexagonal contour (Fig. 1B). Our oblique sections revealed the characteristic porous structure of the interlamellar membranes (Fig. 1C). Tablets consisted of an aggregation of globular particles measuring between 20-50 nm (Fig. 1D–F). The irregular topography became particularly evident with secondary electrons (SE) (Fig. 1E), whereas it remained masked in TEM (Fig. 1B–D). Similar nanoparticles were also found over the interlamellar membranes (ILMs) (Fig. 1D–F), and are likely the remains of hillocks cut during sample preparation. Hillocks, i.e. the protrusions (20–100 nm) of the tablet surface across the pores of the ILM (Supplementary Fig. 1), maintain crystallographic coherence with the rest of the tablet^20^. HRTEM highlights that the cores of these globular particles both from the tablet interior and/or the hillocks were crystalline nanodomains (average size 30 nm) embedded in an amorphous matrix (5–10 nm in thickness; Fig. 1G-I). Some forming tablets also presented amorphous edges, which crystallized under the electron beam (in much the same way as reported by Nassif *et al*.^11^; Supplementary Fig. 2). In other instances, edges were fully crystalline.

**Figure 2:**
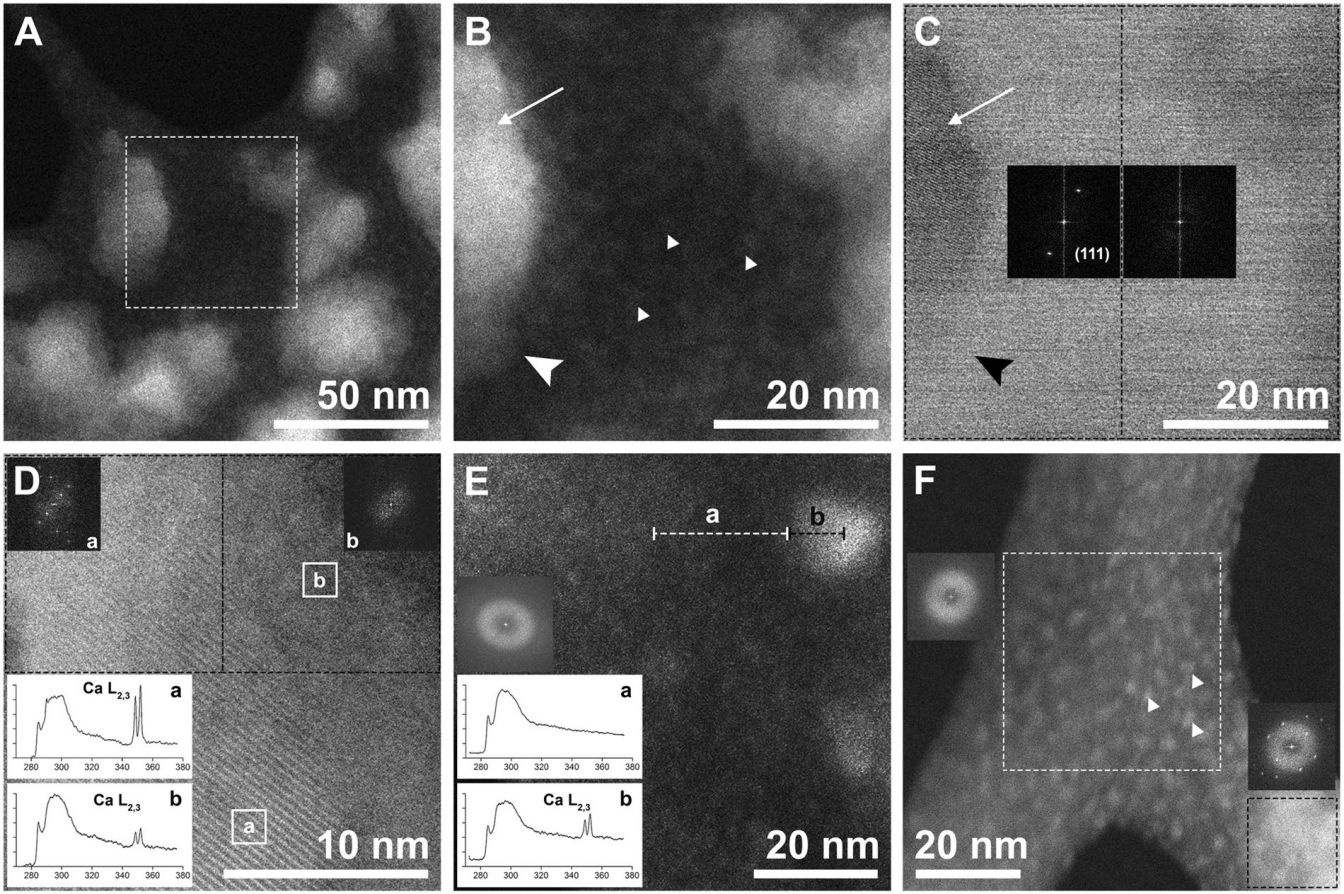
Nanoparticle crystallinity and composition. (**A**) Crystalline nanoparticles (rests of tablets and/or hillocks) over the interlamellar membrane (organic amorphous material) (HAADF). (**B**) Closer view of the area framed in A. The highest contrast (brightest area) corresponds to the core of the particle (long﻿ arrow). The contrast fades towards the lower edge of the same particle (large arrowhead). The nanoparticle to the right presents an intermediate contrast. Densely scattered type- one nanoparticles (2–5 nm) (small arrowheads) with some contrast appear covering the organic amorphous material. (**C**) Bright-field (BF) STEM image of B. The particle to the left has a crystalline core (long﻿ arrow) (indicated by lattice fringes and reflections in the FFT analysis). Its lower part (big arrowhead) does not show lattice fringes. Dashed rectangles indicate the areas where the FFTs come from. (**D**) Nanoparticle showing the crystalline area (lattice fringes and reflections) on the left and the surrounding amorphous material on the right (HAADF). Squares with dashed lines indicate the areas where the FFTs come from. Two EELS spectra from crystalline (a) and amorphous (b) areas are shown (background removed, deconvolved, normalized and smoothed). The areas where the spectra where obtained are indicated by solid squares. (**E**) Line scan EELS performed across the organic membrane (dashed line a) and a type-two particle (dashed line b). Only the particle with high contrast (12 nm in size) presented an intense calcium signal (b) (background removed, deconvolved,normalized and smoothed). The FFT from the whole image shows that no crystalline structure exists. (**F**) Densely scattered type-one nanoparticles (2–5 nm) (small arrowheads) over the organic membrane. FFTs show that nanoparticles have amorphous structure, unlike the bright lower right area, which has crystalline structure.

The examination of nanoparticles spread over the ILMs using High Angle Annular Dark Field (HAADF) imaging allowed us to differentiate the amorphous coating from the organic (also amorphous) background, which remained concealed in TEM (Supplementary Fig. 3). HAADF enhances the contrast between materials of different composition (different average atomic Z number) (Fig. 1F). For the same average Z, variations in contrast are due to density or thickness changes^21^. Accordingly, CaCO_3_ nanoparticles showed an intense contrast (bright areas) with respect to the organic background (Fig. 2A). The highest contrast (Fig. 2B) corresponded to the crystalline core identifiable by lattice fringes (Fig. 2C). Cores were bordered by non-crystalline areas with intermediate contrast (Fig. 2B–D) that faded gradually towards the edges (Fig. 2C,D). Electron Energy Loss Spectroscopy (EELS) confirmed the same qualitative composition for both the crystalline cores and the amorphous rims (Fig. 2D), with the intensity of the calcium L_2,3_-edge being significantly higher in the crystalline cores.

Besides the sectioned hillocks, there were two other types of nanoparticles scattered over the organic amorphous material: type one, 2–5 nm in size (Fig. 2B,F), and type two, 10–20 nm (Fig. 2E). Both of these nanoparticle types returned a calcium signal (Fig. 2E) and had a non-crystalline structure (Fig. 2E,F).

**Figure 3:**
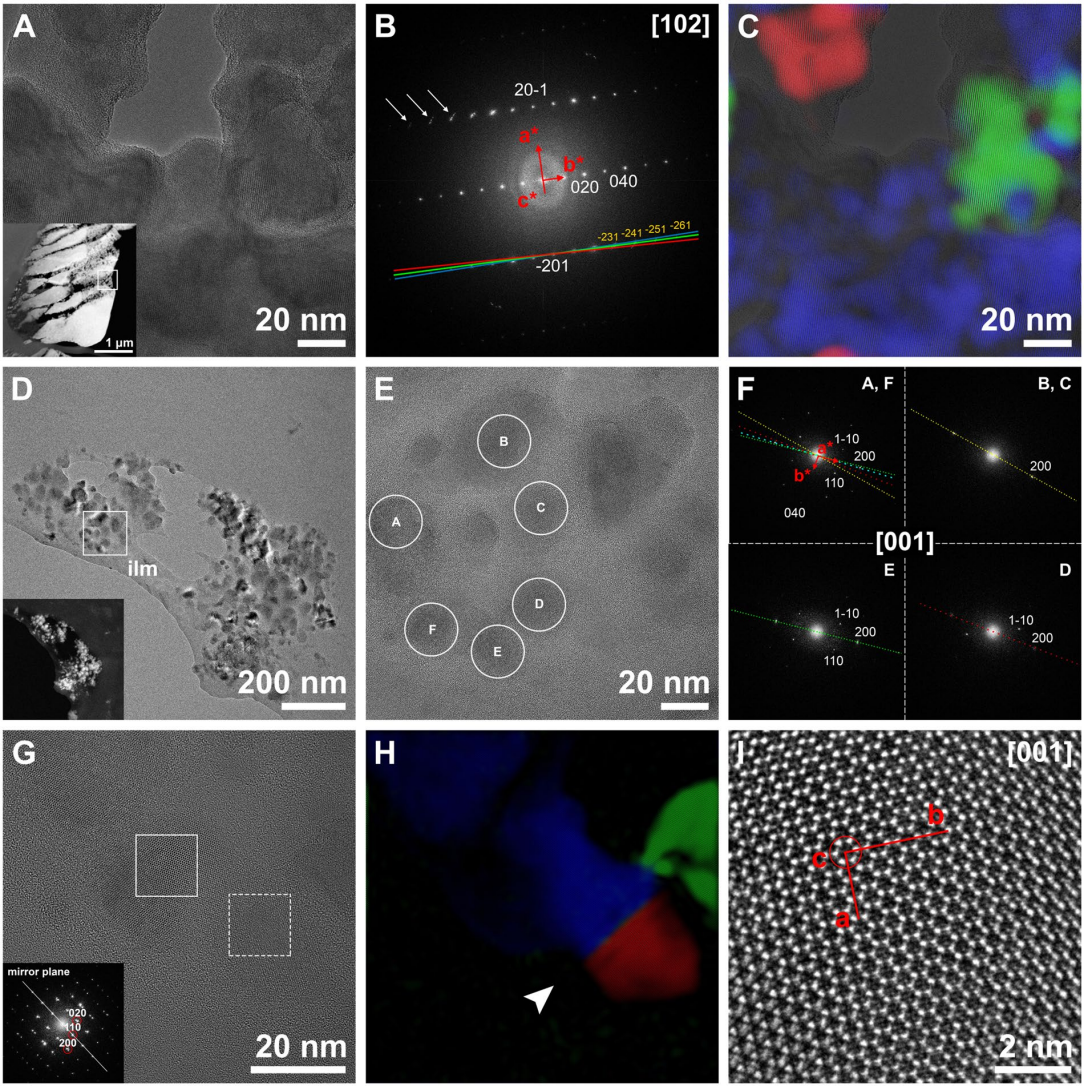
Orientations of crystalline nanodomains. (**A**) TEM image of crystalline nanodomains embedded in amorphous material. Inset: general view of the tablet with the location of the nanodomains. (**B**) FFT analysis of A. The presence of small arcs in the FFT (arrows) indicates slight misalignments between nanodomains. Colours of lines indicate the reflections used for the colour reconstruction in C. (**C**) RGB colour model built using the reflections corresponding to the planes ($\bar{2}$31), ($\bar{2}$41), ($\bar{2}$51) and ($\bar{2}$61) shown in B. Overlap of the original TEM image shows that crystallographically coherent nanodomains are separated by amorphous material. (**D**) Incipient tablet showing crystalline nanodomains immersed in amorphous material. The position of the interlamellar membrane is indicated (ilm). Inset: HAADF of the same area. (**E**) HRTEM of the area framed in D. Crystalline nanodomains over the interlamellar membrane. (**F**) FFT analysis of the nanodomains encircled in E (with the same letters). They are all indexed as aragonite along the [001] zone axis. The nanodomains are slightly tilted, which results in the attenuation or even disappearance of some reflections. In each FFT, the *a-*axis is marked with a coloured dotted line. The overlap of all lines (upper left FFT) shows the misalignment of *a*-axes between nanodomains. (**G**) HRTEM image of three different nanodomains over an organic membrane. Two of them show a twin relationship. Inset: FFT of the twin domain (area framed with dashed line). (**H**) Colour reconstruction of the three different domains. A (110) twin plane (arrowhead), characteristic of aragonite, separates the blue and red nanodomains. The green nanodomain is in slight contact with the blue one, with a misorientation of 5.8° around the *c*-axis. (**I**) Enlargement of the area framed in G (solid line, blue nanodomain) indicating the crystallographic orientation. The view is from the *c*-axis.

Indexing of crystalline nanodomains (30–50 nm) surrounded by amorphous matter of the tablet interior corroborated their aragonite nature (error <5%). No other polymorph was detected. FFT of the image covering several nanodomains in Fig. 3A returned short arcs instead of sharp points, which result from the addition of slightly misaligned reflections (Fig. 3B). By selecting the signals of the misaligned planes from the FFT and inverting the FFT function, we located the source areas of these signals. In this way, it became evident that the signals came from adjacent, slightly misaligned (1.7° and 1.9°) nanodomains (Fig. 3B). Remarkably, the nanodomains were not oval, but had highly irregular contours that connected with and even overlapped one another (Fig. 3C). A case similar to that in Fig. 3A–C is shown in Supplementary Fig. 4.

When indexing could be made exactly along the [001] zone axis, we appreciated that the crystalline nanodomains (Fig. 3E) had their *c*-axes co-aligned, but they were slightly misaligned in the *a-b* plane (Fig. 3F). An example of hillocks adhering to ILMs is shown in Fig. 3D–F. Some nanodomains presented twin relationships (Fig. 3G–I).

**Figure 4:**
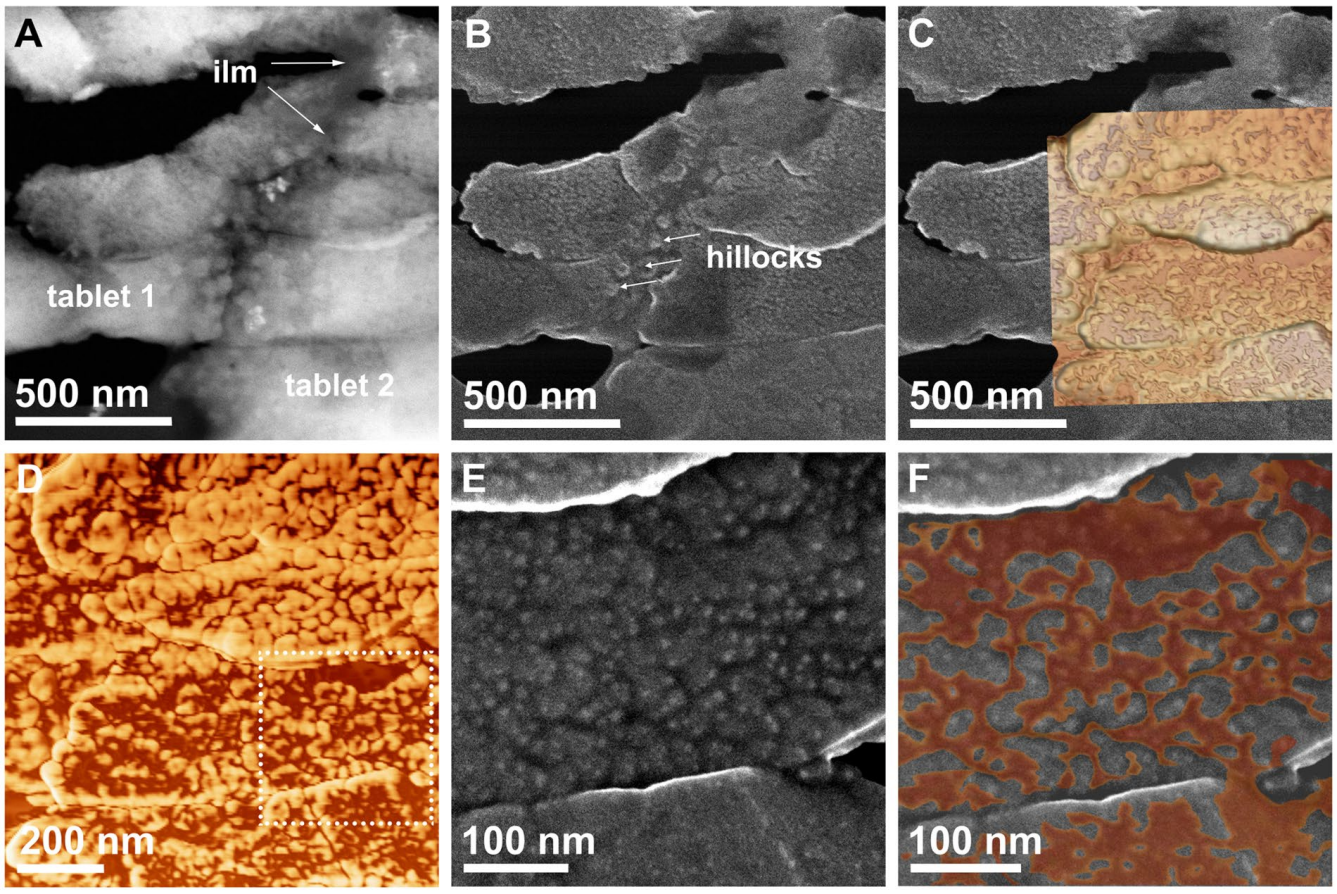
SE and AFM analysis of the same area. (**A**) HAADF image of two tablets and the intermediate interlamellar membrane (ilm) (darker material). (**B**) SE image of the same area. The smoothest area corresponds to the interlamellar membrane and some hillocks are visible at the boundary between the two tablets. (**C**) SE image with partially superposed AFM image (topographic + phase mode). (**D**) AFM phase image emphasizing the globular aspect of the structure. The more adhesive material (dark-brown areas) overlies the crystals (light-brown areas). (**E**) SE image of the area framed in D. The white dots are particularly steep areas, whereas darker areas tend to be horizontal. (**F**) Overlap of the darker areas of the AFM phase (brown) and SE (dark grey) image.

We performed both AFM and S/TEM on single tablets (Fig. 4), with both revealing the globular texture typical of nacre^17,22,23^ and other biominerals^10,24^. AFM phase contrast images (which enable the detection of variations in adhesion, friction and viscoelasticity^25^; see explanation in Supplementary Fig. 5) revealed two different materials (Fig. 4D): one with more adhesive strength (low contrast), previously assumed to be either organic material^17,22^, or a mixture of organics and ACC^26^, and a second, stiffer material, assumed to be the crystalline phase by the same authors. The topographic images showed that the low-contrast layer extends over the stiffer material in the form of discontinuous pellicles (with variable thicknesses, between 2-10 nm, n = 10).

Observation of exactly the same areas by S/TEM (Fig. 4A–C) provided complementary information. The topographic contrast generated by SE in STEM is basically surface-tilt contrast: SEs created on an inclined surface or close to a surface step have an increased probability of escaping, thus producing a higher brightness/contrast^27^. The irregularities observed with SEs (Fig. 4E) were less pronounced in the AFM images, but they correlated well with the topographic profiles, that revealed a very irregular surface. The overlap of both the SE and the low-contrast phase of the AFM images (Fig. 4F) showed that the distribution of the latter coincided relatively well with the flat (low-contrast, dark grey) areas observed in SE images.

**Figure 5:**
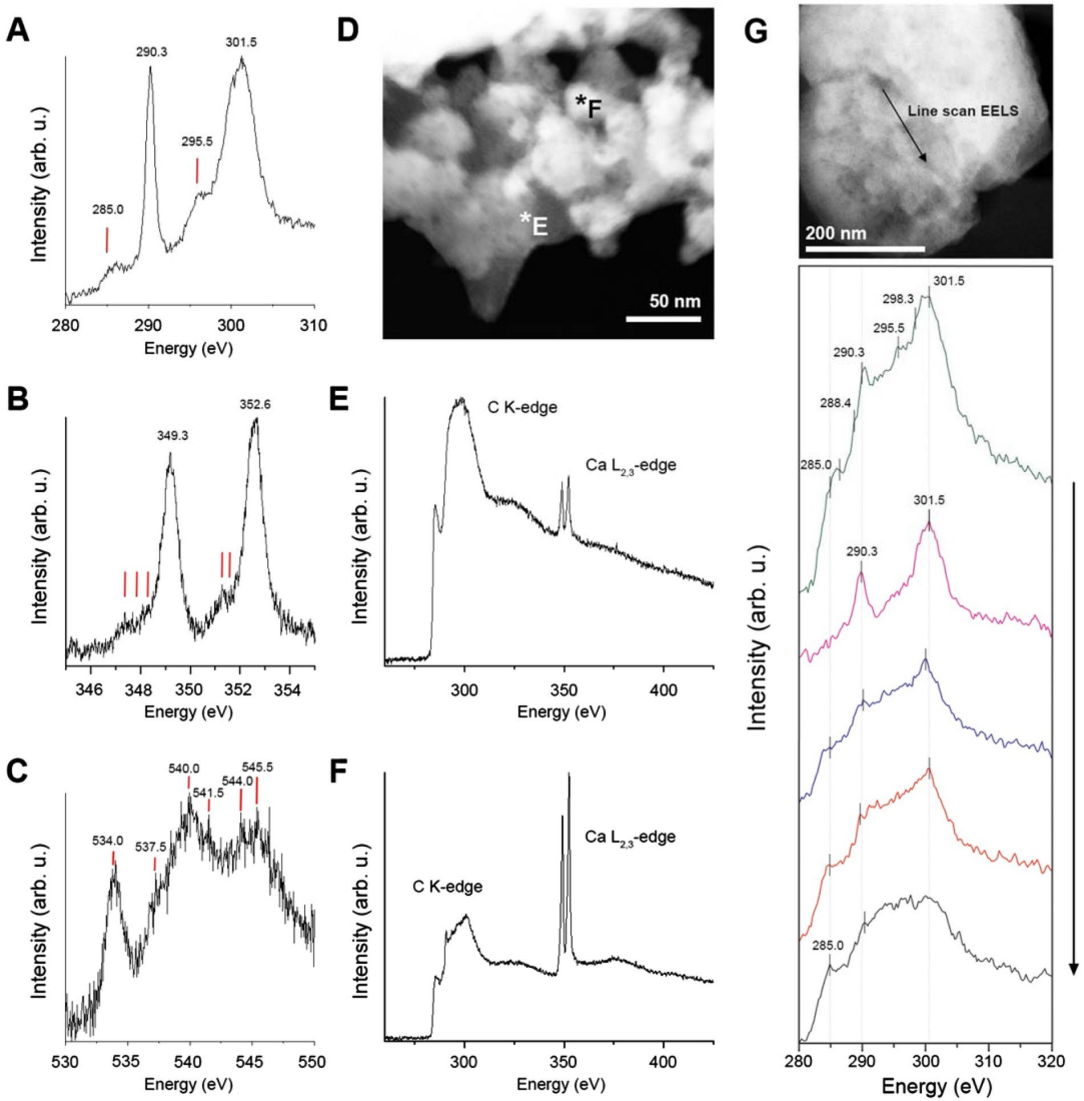
EEL Spectroscopy from incipient nacre. (**A**) The C K-edge shows two prominent peaks at 290.3 and 301.5 eV, indicating high crystallinity, but a weak hump at 285 eV that reflects the presence of organic material. (**B**) Ca L_2,3_ edge. (**C**) O K-edge. For **A**,** B** and **C**, background was removed before each edge. The spectra are normalized and the intensity is expressed in arbitrary units (arb. u.) for comparison. No smoothing was applied. (**D**) HAADF image showing the points where spectra E and F were taken. (**E**) C K-edge and Ca L_2,3_-edge from amorphous area. (**F**) Same edges from an area where crystalline and amorphous materials overlap (background removed and normalized). (**G**) STEM-EELS line scan showing progressive changes in the C K-edge from crystalline to amorphous areas. The top image depicts the segment (142.7 nm) where the line scan was performed (arrow indicates the direction of measurements). A total of 25 spectra (C K-edge) were taken (each 5.75 nm, dispersion 0.25 eV) and averaged (5 single spectra added) to reduce the noise. Background was removed before the C K-edge, and plural scattering was corrected. No smoothing was applied. Vertical lines represent locations of peaks described in Table S1. All spectra were acquired in vacuum (with no organic membrane or support underneath).

EELS performed in crystalline and amorphous domains revealed differences in chemical bonding (Fig. 5). The C K-edge ELNES (Electron Energy-Loss Near-Edge Structure) spectra of crystalline areas (acquired in vacuum) showed two main peaks at 290.3 eV and 301.5 eV (Fig. 5A) that are unambiguously indicative of the CO_3_^−2^ groups^28–30^. The peak at 290.3 eV corresponds to C1s → π* transitions, whereas the peak at 301.5 eV arises from C1s → σ* transitions of carbon-oxygen bonds of carbonate CO_3_^−2^ groups^29,31^. Minor features related to π* transitions of carbon-oxygen bonds, at 294.2 and 298.3 eV^31^, were identified in only some spectra, with the former shifted to higher energies (295.5 eV) (Fig. 5A, Supplementary Fig. 6). Thus, the C K-edge is typical of carbon in carbonate groups in the crystalline areas.

In amorphous areas, the C K-edge showed an initial peak at 285 eV and a second broad feature at 290 eV, typical of amorphous carbon (Fig. 5D, E). In areas where both crystalline and amorphous materials coexist (Fig. 5D, F), the spectrum was a linear combination of the spectra of amorphous carbon and carbon in carbonate form^32^.

Three pre-peaks at 285.0 (±0.3), 287.5 (±0.3) and 288.4 (±0.3) eV (Supplementary Fig. 6), with different relative intensities depending on the sampled area, were identified. The 285.0 and 287.5 eV peaks arise from C1s → π* in C=C and from C1s → σ* in C–H in organic molecules, respectively^33,34^. The 288.4 eV peak results from C1s → π* in C=O of carboxyl groups of organic molecules^31,33,35^. In crystalline areas only the 285.0 eV peak appears (Fig. 5), whereas in non-crystalline areas we recorded all three peaks (Supplementary Fig. 6). This indicates that although organic material is present in both phases, it is more noticeable in the amorphous phase.

The calcium L_2,3_-edge ELNES spectrum is characterized by two main peaks at 349.3 and 352.6 eV (Fig. 5B). In the crystalline areas the presence of three and two minor features before the Ca L_3_- and the Ca L_2_-edges respectively indicates an aragonite nature^13^. Although we were unable to record the typical ACC profile (see methods), the Ca L_2,3_-edge in the amorphous areas showed significantly less intensity (i.e. less crystallinity) (Fig. 2D).

The oxygen K-edge ELNES spectra in the crystalline areas are characteristic of aragonite, with a main peak at 534 (Fig. 5C) that results from O1s → π* transitions from the C=O bonds^34,36^, followed by a shoulder at 537.5 eV. Two double peaks are defined at 540/541.5 eV, associated with the O1s → σ* transitions of the carbonate CO_3_^−2^ group^34^ and at 544/545.5 eV, corresponding to O1s → σ* transitions from the C=O absorptions^36^ (assignments of peaks are summarized in Supplementary Table 1). In the amorphous areas, the O K-edge lost any fine structure and showed only a broad resonance.

In the transition to the amorphous areas the three edges presented a progressive reduction in the intensity of the peaks. The peaks from the C K-edge graded to a single broad shoulder (Fig. 5G), typical of amorphous carbon. The Ca L_2,3_-edge diminished drastically in intensity, and the O K-edge lost any fine structure and showed only a broad resonance.

## Discussion

To test the hypothesis concerning the formation of the mature nacre through an amorphous transient phase, we selected incipient tablets of subadult gastropods. Our TEM study demonstrated that the tablets are composed by globules with a partially crystalline structure: a crystalline core (~30 nm) and an amorphous contour (~5–10 nm). The amount of amorphous material in developing crystals is significantly higher than that found in mature crystals^14^, in line with the results of DeVol *et al*.^13^, which suggests that the amount of amorphous material dwindles with the advance of the crystallization and the maturation of the tablet.

Within individual tablets, the indexing of adjacent crystalline nanodomains connected by amorphous material showed a minor misorientation (in the range of 2°). In particular, the *c*-axes showed the highest degree of co-alignment. The quantification of the misalignment of the *c*-axes of mature nacre tablets of *Mytilus edulis* with low acceleration voltage electron backscatter diffraction (EBSD) gave a mean angular spread in the order of 2° FWHM inside individual tablets^37^, in agreement with our present results. The RGB colour reconstruction of the nanodomain orientation (Fig. 3C) clearly showed that the sectional outlines of the crystalline nanodomains are not simply spherical or oval, but complex, digitiform. The fact that separate digitiform domains were in exactly the same orientation indicates that they must connect in the third dimension. Apparently misoriented nanodomains were also found in our samples. Nevertheless, the impossibility of indexing them as aragonite or any other calcium carbonate polymorph has forced us to disregard them. Their presence might be related to sample preparation or beam damage (i.e. transformation into CaO), to which biogenic calcium carbonate is highly sensitive^38^. The finding of similarly misoriented nanodomains in the nacre of the bivalve *Perna viridis* led Zhang and Xu^12^ to propose a formation mechanism based on the aggregation of completely misoriented nanodomains that afterwards realign by oriented attachment. Our data do not support this hypothesis. Nevertheless, we might consider the unlikely possibility of differences related to the independent origins of nacre in the four nacre-forming molluscan classes (gastropods, bivalves, cephalopods and monoplacophorans)^39^.

Both organic matter and ACC have amorphous structures, making it unfeasible to differentiate them under traditional TEM, unless ACC is transformed by irradiation. Accordingly, the amorphous rims of nanoparticles were characterized by HAADF. The lack of lattice fringes and the recorded calcium content indicate that it is ACC. The lower contrast with respect to the crystalline core is likely to be due to its lower density. The density of ACC (ρ _ACC_ = 1.62 g·cm^−3^)^40^ is significantly lower than that of the crystalline phase (ρ_aragonite_ = 2.93 g·cm^−3^).

At the transition from crystalline to amorphous areas, the C K-edge changed from multiple sharp peaks to a broad hump (Fig. 5D-F). Three pre-peaks at the C K-edge (285.0, 287.5 and 288.5 eV) appeared in both crystalline and amorphous material and are undoubtedly related to the presence of organic matter. Chitin and lipids can contribute to these peaks^41,42^, but their low content in the organic fraction of nacre (~7% chitin^43^; 0.54% lipids^44^) leads us to believe that mostly proteins are responsible for these signals.

In the O K-edge, the intensity of the 534 eV peak varies with the degree of crystallinity^36^. The intensity of the 534 eV peak in mature nacre is markedly higher than for the rest of the features^45^. The weak 534 eV peak found in our samples indicates poor crystallinity.

In conclusion, our HRTEM and the ELNES analyses of the amorphous phase in incipient nacre tablets indicate that the phase that surrounds and partially covers the crystalline phase is composed of a mixture of ACC and organic matter (mainly proteins). This phase was previously suggested to be either organic^17,22^ or a mixture of organic macromolecules and ACC^26^, but without conclusive evidence. We can ascertain that the latter is the case. This same amorphous phase, when observed under AFM, has a higher adhesion strength.

The presence of biogenic macromolecules occluded within the ACC stabilizes it against dissolution or rapid crystallization^46–48^. Different kinds of additives have been identified as stabilizers, with amino acids being among the molecules that permanently stabilize ACC^44^. Proteins have been demonstrated to inhibit ACC·H_2_O dehydration^49^, allowing the persistence of ACC·H_2_O-rich nanoparticles.

In nacre, the continuity of the mineral bridges from one tablet to the next^50^ would allow the propagation of the crystal lattice. A neat crystallization front is absent. Conversely, we have observed complex, digitiform fronts that in 3D should branch off throughout the interior of the material. Transformation from amorphous to crystalline would happen concomitantly along this large-surface crystalline-amorphous interface. Concomitant transformation at different sites was implied by Addadi *et al*.^47^ to occur also in the sea urchin larval spicule.

As the tablets mature and ACC crystallizes, some of the organic macromolecules may be occluded within the crystal lattice, while others would be expelled by the crystallization force and would tend to concentrate in progressively shrinking ACC areas. Their accumulation might “poison” the crystallization process^51^ and permanently stabilize the residual organic-rich ACC, which would accumulate around crystalline globules^26^. These would remain as pellicles around crystalline globules (commonly observed as low areas contrast in AFM phase images).

**Figure 6:**
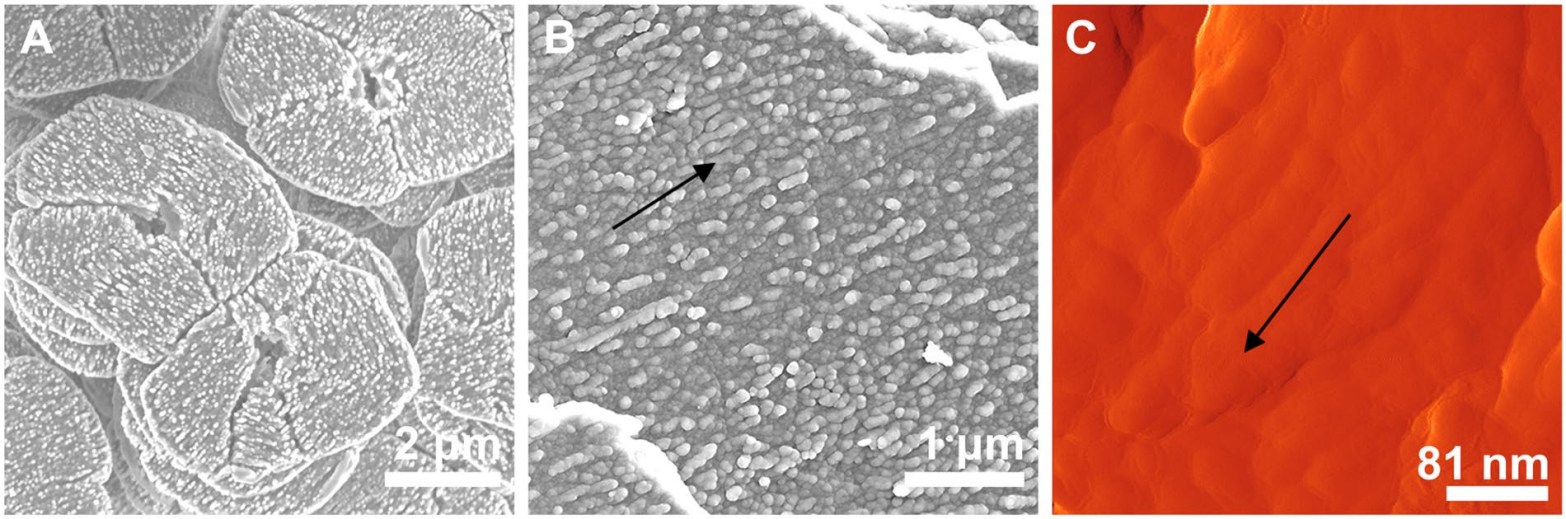
Fused and aligned nanoglobules in nacre. (**A**) Gastropod *Gibbula cineraria* nacre tablets, etched with Mutvei’s solution, SEM view. (**B**) Cephalopod *Nautilus pompilius* septal nacre, bleached for 3 min, SEM view. (**C**) *Pteria hirundo*, treated with protease (0.2 g/ml, 1 h), AFM amplitude image.

This model entails a noteworthy conclusion: our observations reveal that the nanoglobules consist of a crystalline core and an amorphous cortex; the crystalline cores can be assimilated to the fingers emitted from the digitiform crystallization front. Accordingly, the observed nanoglobular structure of nacre is due to the presence of precursor ACC but is modulated during the progressive process of transformation of ACC into aragonite. Our results match those of Checa *et al*.^52^, who found that the nanoglobules in nacre elongate and/or align in parallel to the *a-*axis of aragonite (Fig. 6), i.e., they are crystallographically controlled features.

Our data are consistent with a particle-accretion mechanism in nacre. The two types of ACC nanoparticles detected on the ILMs (type one, 2–5 nm, and type two, 10–20 nm) are in the size range of the aggregation units described in synthetic systems^53,54^ and it is likely that they are the first ACC intermediates. Hence, the sizes of the nanoglobules do not necessarily represent the sizes of the initial aggregation particles, contrary to what was implied by some authors^48^.

Two hypotheses have been proposed for the transformation of ACC into the crystalline phase. First, transformation was hypothesised to occur via secondary nucleation^55,56^ (but see^14^ for the inadequacy of the term), whereby both phases are in contact and undergo a solid-state transformation^57^. Nevertheless, as described above, the final nanostructure is modulated by the transformation of ACC into aragonite in the presence of organic molecules, which are mobilised towards the contours of grains. This is only possible through the interface-coupled dissolution-precipitation process^15,58,59^, which implies the existence of a fluid phase (partly resulting from H_2_O released during the transformation of ACC into aragonite). In this way, reshaping of the overall nanogranular structure of grains takes place by regrowth of the crystalline phase (which can proceed via a classic layer-by-layer mechanism), until the nanograins acquire their final shapes, sizes (up to one order of magnitude bigger than the precursor ACC nanoparticles) and arrangements. This mechanism leads to pseudomorphs, thereby preserving the nanogranular structure imprinted during the early stages of nacre (Carlos Rodríguez-Navarro, personal communication, 2017). Regrowth was experimentally demonstrated in calcite^58^. Given the similarity in nanostructure of nacre with most biominerals, it is likely that all of them followed a similar ACC-to-crystalline transformation route.

## Methods

Specimens of the nacreous gastropod *Phorcus turbinatus* were caught alive in La Herradura (36°43′44″N, 3°43′35″W), on the coast of the province of Granada (S Spain), *in vivo* frozen in liquid nitrogen and stored in a dewar flask. They were freeze-dried the day afterwards: the initial temperature (−170 °C) was raised slowly at an average pressure of 75 mTorr (Flexi-Dry MP); at the end of the drying cycle (2 days), when room temperature was reached (20 °C), the specimens were removed and stored at 4 °C. This process implies the removal of unbound water (sublimation phase or primary drying). The extraction of bound water requires a second phase (secondary drying or desorption) at relatively high temperatures (25° to 40 °C)^60^.

Specimens were cut with a diamond saw and pieces from the nacre growth area were selected for embedding with Embed 812 (EMS), increasing the proportion of embedding medium to pure ethanol in three steps (1:2, 1:1 and 2:1). Osmication, *en bloc* uranyl acetate, and post-staining were avoided to prevent water contact and the addition of heavy metals that can interfere with analytical techniques.

Ultramicrotome (PowerTome X, Boeckeler) slices were cut as parallel as possible to the internal shell surface. With the progress of the diamond knife the crystals broke into fragments with parallel edges, but the nanostructure remained in its original condition. The slices were laid on copper grids with lacey carbon to stabilize them under the electron beam. For imaging and elemental analysis, we used a double Cs corrected JEOL JEM-ARM200F TEM (Fritz Haber Institute of the Max Planck Society, Berlin) equipped with a cold-field emission gun, an energy dispersive X-ray detector (JEOL), a Gatan Imaging Filter (GIF Quantum), and a Gatan UltraScan 4000 camera.

The nanodomains were indexed by Fast Fourier Transform (FFT) analysis, using the DiffTools script package^61^ for Digital Micrograph (Gatan Inc.). The refined aragonite parameters of Caspi *et al*.^62^ were used to construct the unit cell in CaRIne Crystallography 3.1^63^.

The *c*-axes of nacre towers rarely coincided with the nanodomain zone axis because sectioning was hardly perpendicular to the growth axis of the nacre towers (i.e. the normal to the section plane), the angles ranging between 0° and 24° for 12 nanodomains from different slices (Supplementary Fig. 7). Due to the sensitivity to electron beam irradiation, tilting of crystalline domains in a zone axis could not be performed. Therefore, we studied the relative orientations of adjacent nanodomains instead (Fig. 3 and Supplementary Fig. 4).

Electron Energy Loss Spectroscopy (EELS) spectra from 250 to 550 eV were recorded using a dispersion of 0.25 eV/channel, with a collection semi-angle β = 27.7 mrad and a 5-mm spectrometer entrance aperture. The energy resolution was ~1.25 eV measured by the full width at the half maximum (FWHM) in the zero-loss peak. For spectra covering two edges (i.e. from 275 to 375 eV), we used a dispersion of 0.05 eV/channel, with a collection semi-angle of β = 20 mrad and a 5-mm aperture. The energy resolution was ~0.65 eV at FWHM in the zero-loss peak. For one edge, we used a dispersion of 0.025 eV, a collection semi-angle of 14 mrad and a 2.5 mm aperture; energy resolution was ~0.5 eV at FWHM. Emission current was set to 5 µA and the acquisition time was optimised to acquire sufficient signal intensity and to limit beam damage. The appearance of a prominent peak at 532 eV in the O K-edge is considered to be an indication of beam damage^64,65^, so the integral dose applied was adjusted to below this threshold.

Only changes in the C k-edge could be tracked, given that the edge is very sensitive to bonding changes even at low-energy resolution. Since differences in the fine structure of the Ca L_2,3_ and O K-edges between ACC and aragonite are subtle^13,36^, a high signal-to-noise ratio is necessary to unequivocally assign spectra to either of them, which results in compromising with the dose applied. In other instances, larger areas were used for data acquisition in order to reduce beam damage, thus precluding the characterization of the fine structure of the nanometric amorphous domains.

Background was subtracted before each edge using power-law fitting and plural scattering was removed when necessary using a Fourier-ratio deconvolution, both available in Digital Micrograph 2.30 software (Gatan Inc.). Plotting of the data and normalization were carried out with OriginPro 8.5 software.

Atomic force microscopy (AFM) was performed directly on the TEM grids. Areas of interest were previously selected by SEM on uncoated samples (Phenom Pro, University of Granada, Spain) and later manually located under the AFM using the X-Y translator of the optical head. Images were recorded in air at room temperature using an AFM (Multimode IIIa Veeco Instruments) of the Centro Nacional de Microscopía Electrónica (Universidad Complutense de Madrid, Spain). AFM was operated in tapping mode while displaying cantilever height, phase, and amplitude signals. Different areas of the sample were scanned using a ~14 × 14 μm^2^ piezo scanner and tips with a nominal radius of 7- 8 nm supported by rectangular cantilevers with a nominal resonance frequency of 320 kHz, and a maximum spring constant of 55- 80 N/m (Bruker TESP). The scan rate was set to about 1 Hz, and 256 to 512 lines per scan were recorded. All AFM images were subsequently analysed using NanoScope Analysis 1.50 (Bruker) and Nanotec WSxM. 4.0^66^.

## Electronic supplementary material

Supplementary Information
