# Supplementary material for: Transformation of ACC into aragonite and the origin of the nanogranular structure of nacre
Source: Sci Rep. 2017 Oct 5;7:12728. doi: 10.1038/s41598-017-12673-0 (PMC5629257; doi:10.1038/s41598-017-12673-0)
Supplement: Supplementary file 1 — Supplementary Information [file 41598_2017_12673_MOESM1_ESM.pdf]

# Transformation of ACC into aragonite and the origin of the nanogranular structure of nacre

Elena Macías-Sánchez[1,2], Marc G. Willinger[3,4], Carlos M. Pina[5] and Antonio G. Checa[1,2]*

[1] Department of Stratigraphy and Palaeontology, University of Granada, Granada 18071, Spain.

[2] Andalusian Earth Sciences Institute (IACT), UGR – CSIC, Avd. de las Palmeras 4, Armilla 18100 Granada, Spain.

[3] Department of Inorganic Chemistry, Fritz Haber Institute of the Max Planck Society, Berlin 14195, Germany.

[4] Department of Colloid Chemistry, Max Planck Institute of Colloids and Interfaces, 14476 Potsdam, Germany.

[5] Department of Crystallography and Mineralogy, Complutense University of Madrid, Geosciences Institute (IGEO) (UCM-CSIC), E-28040 Madrid, Spain

Supplementary Figures 1 to 7 and Supplementary Table 1

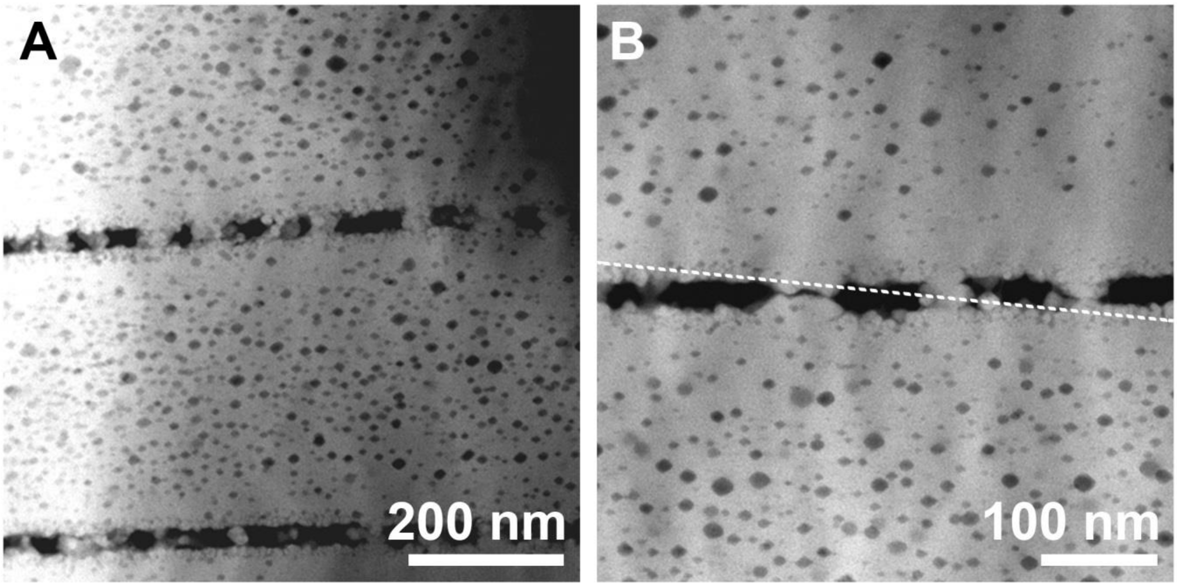


**Supplementary Fig. 1. Position and aspect of hillocks.** A) Three adjacent tablets of the same column showing the intervening hillocks and the typical porous structure of nacre tablets (HAADF). B) Hillocks protrude from both tablets through pores of the interlamellar membranes until they impinge on each other. Although there is physical separation between the two tablets, the crystalline lattice has the same orientation. The dotted line indicates a hypothetical cutting plane through the interlamellar membrane and the hillocks.

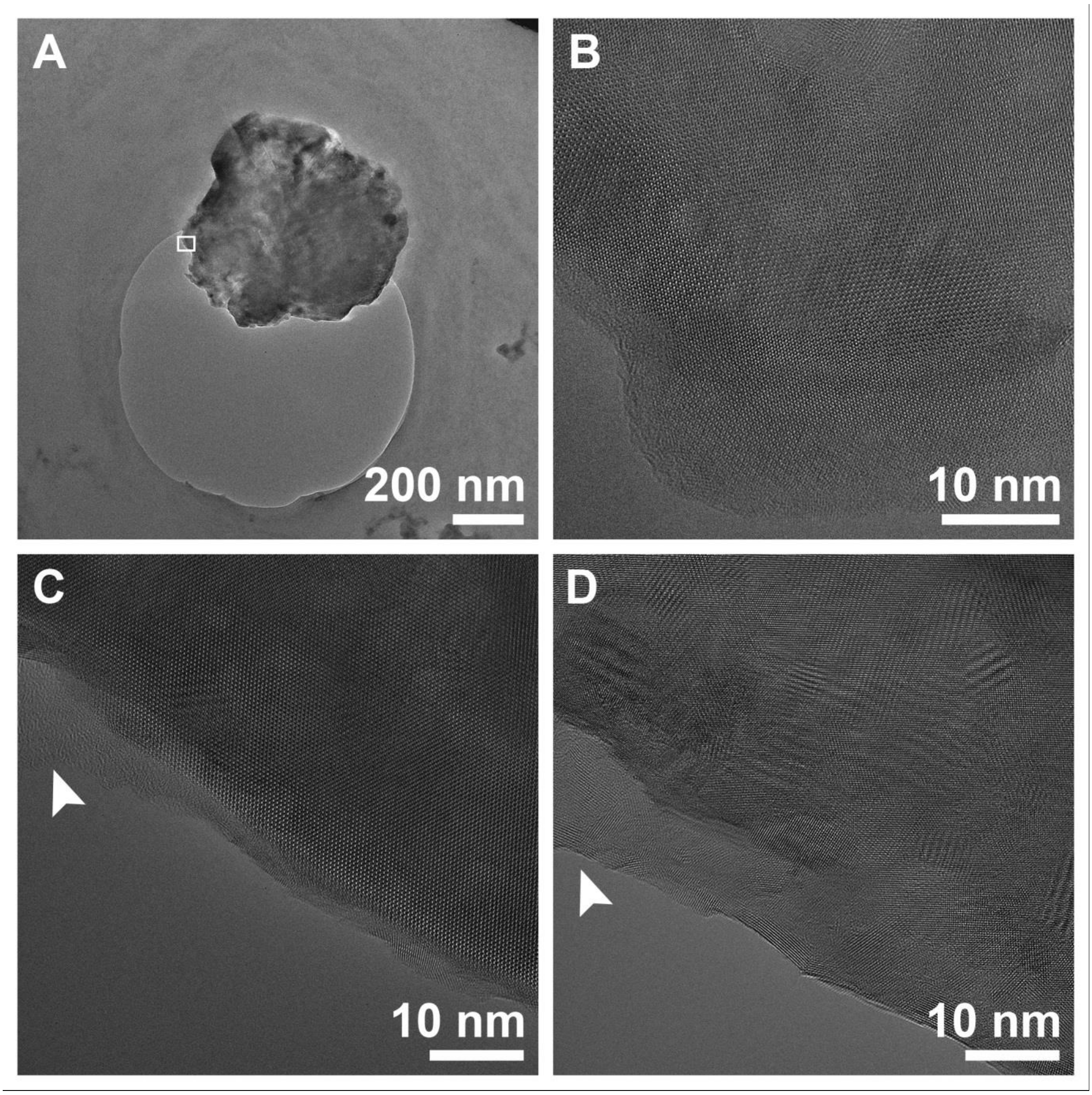


**Supplementary Fig. 2. Amorphous border in an incipient nacre tablet.** A) Overview of the tablet. B) Magnification of the area framed in A, showing the amorphous edge (2-5 nm) that surrounds the crystalline area. C, D) Amorphous edge before and after electron beam irradiation. After irradiation, the amorphous border becomes crystallized (acceleration voltage 200 kV, emission current 10 µA).

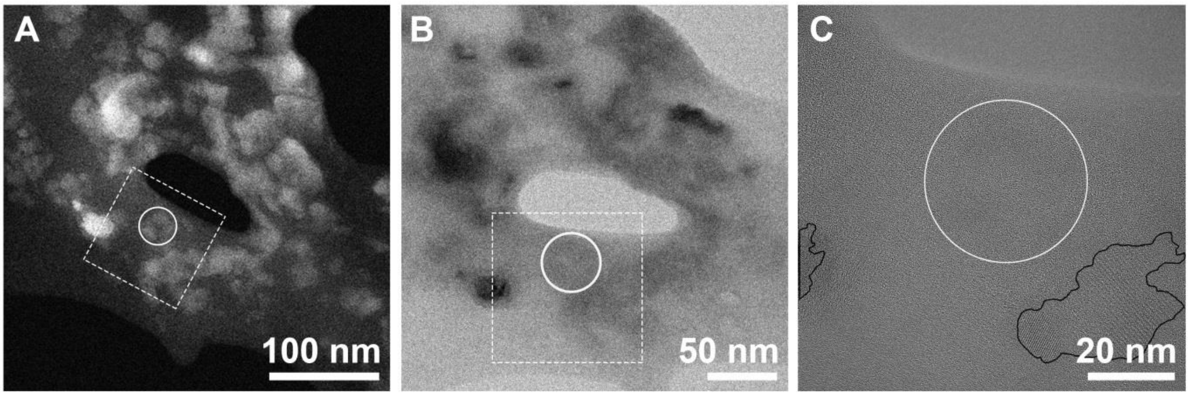


**Supplementary Fig. 3. Image contrast comparison of ACC over an interlamellar membrane.** A) Interlamellar membrane with calcium carbonate particles on its surface (HAADF). The highest contrast areas (white) have crystalline structure, whereas the lowest contrast areas do not. The circle encloses a low-contrast area. B) TEM image (using an objective aperture to enhance the contrast) of the same area. The crystalline areas are recognizable due to their high contrast (black). C) HRTEM from the area framed in A and B. Crystalline areas, recognisable by lattice fringes, are outlined in black. The encircled area, as well as its surroundings, are completely amorphous. ACC cannot be distinguished from the organic background in HRTEM because of their amorphous structures.

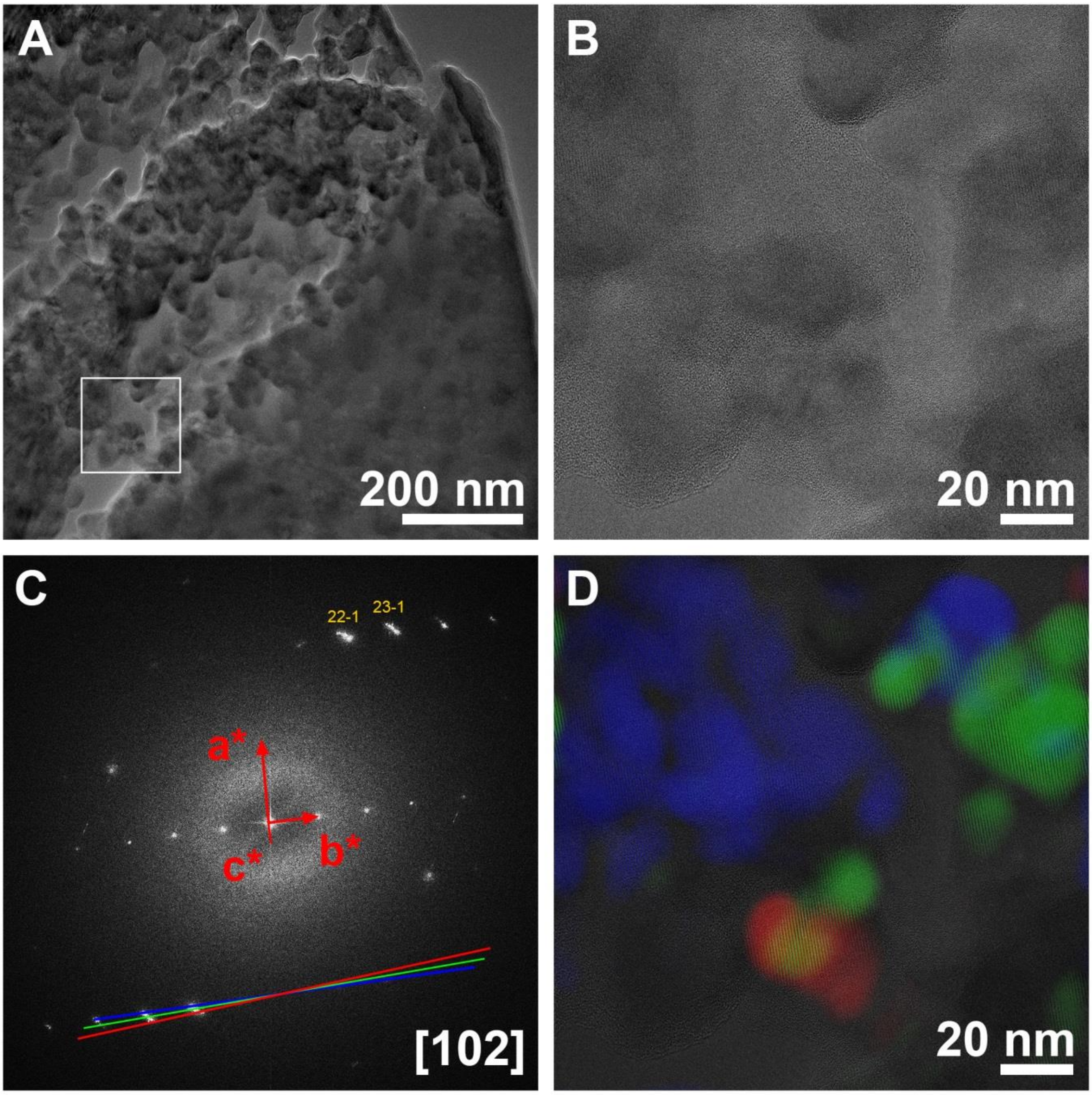


**Supplementary Fig. 4. Orientation of crystalline nanodomains.** A) General view of an incipient tablet where it is possible to see the agglomeration of globular nanoparticles. B) Enlargement of the area framed in A. Crystalline nanodomains are surrounded by amorphous material. C) FFT from the complete area in B. The particles are oriented along the [102] zone axis, but the sample is slightly tilted, giving an incomplete diffraction pattern. The small arcs indicate a slight misalignment. D) The RGB reconstruction using the reflections of the $(22\bar{1})$ and $(23\bar{1})$ planes defines adjacent nanodomains misaligned by 1.2° from each other.

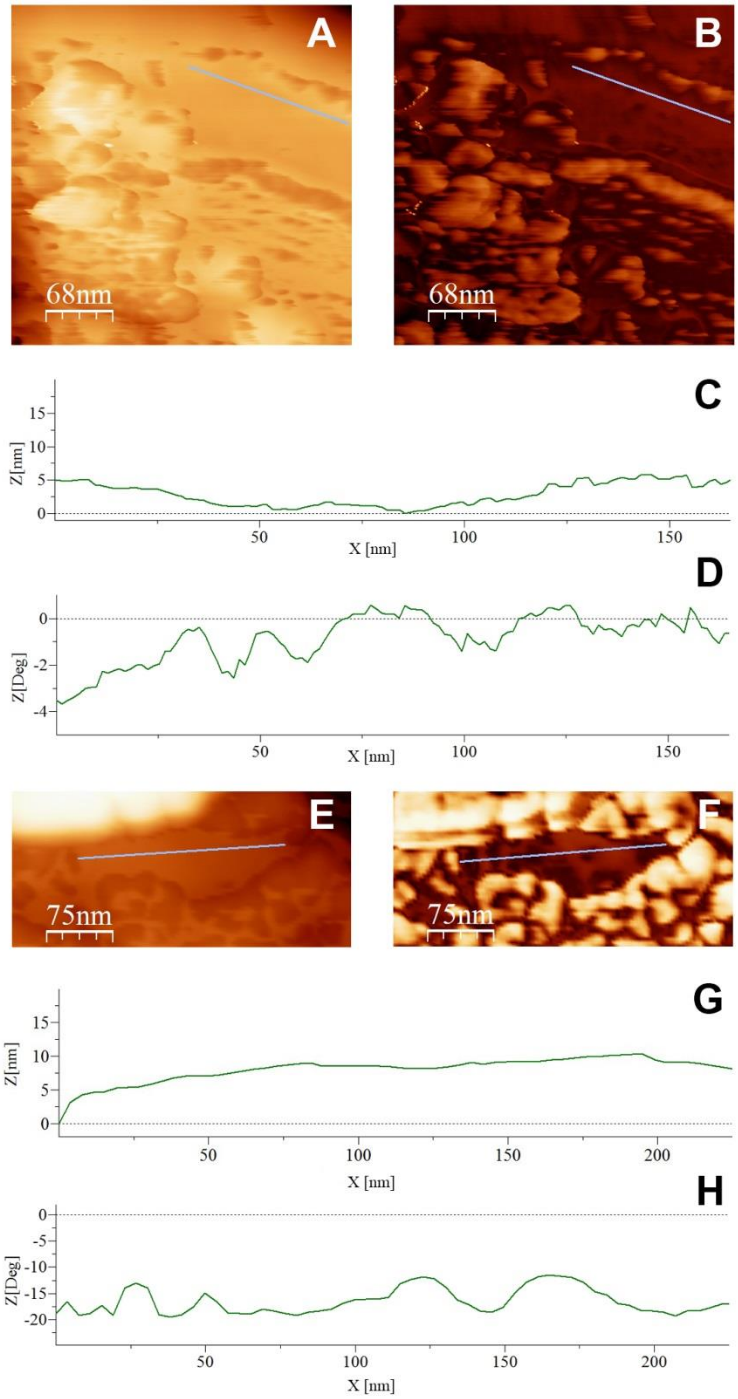


**Supplementary Fig. 5. Differential information provided by tapping mode height and phase AFM images of nacre tablets.** Two examples (A, B and E, F) of relatively flat areas where the topographic and phase contrasts do not coincide are provided. Height (A, E) and phase contrast (B, F) images are shown separately, together with identical profiles for height (C, G) and phase (D, H). Comparison between height and phase profiles (C-D, and G-H) demonstrates that the influence of the topography on the phase signal is negligible.

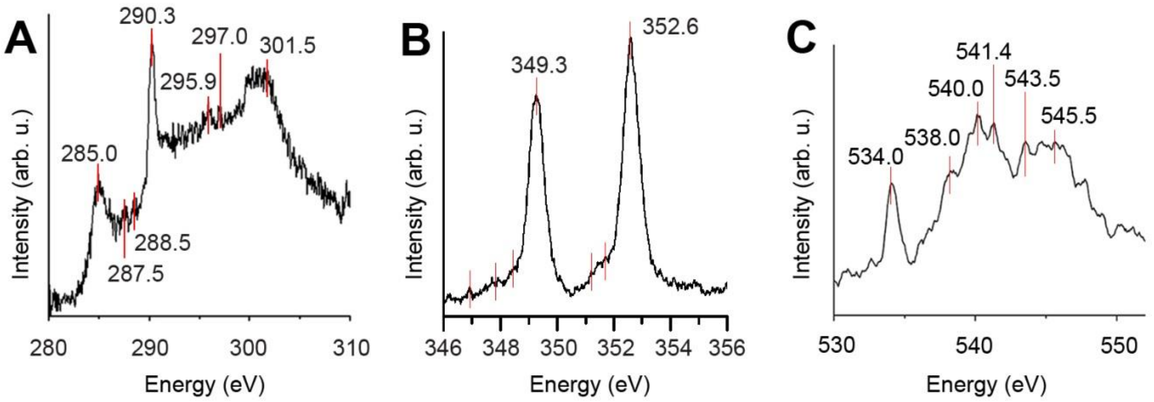


**Supplementary Fig. 6. EEL Spectroscopy.** A) Carbon K-edge. The three pre-peaks at 285.0, 287.5, and 288.5 eV, characteristic of organic material, are visible. B) Calcium $L_{2,3}$-edge. The minor features that indicate the aragonite nature are marked with red bars. C) Oxygen K-edge characteristic of aragonite. All the peak positions denoted by vertical lines are commented on Table S1. Emission current 5 µA, dispersion 0.025 eV. Savitzky-Golay smoothing was applied to both the C K- (10 pts) and the O K-edges (50 pts).

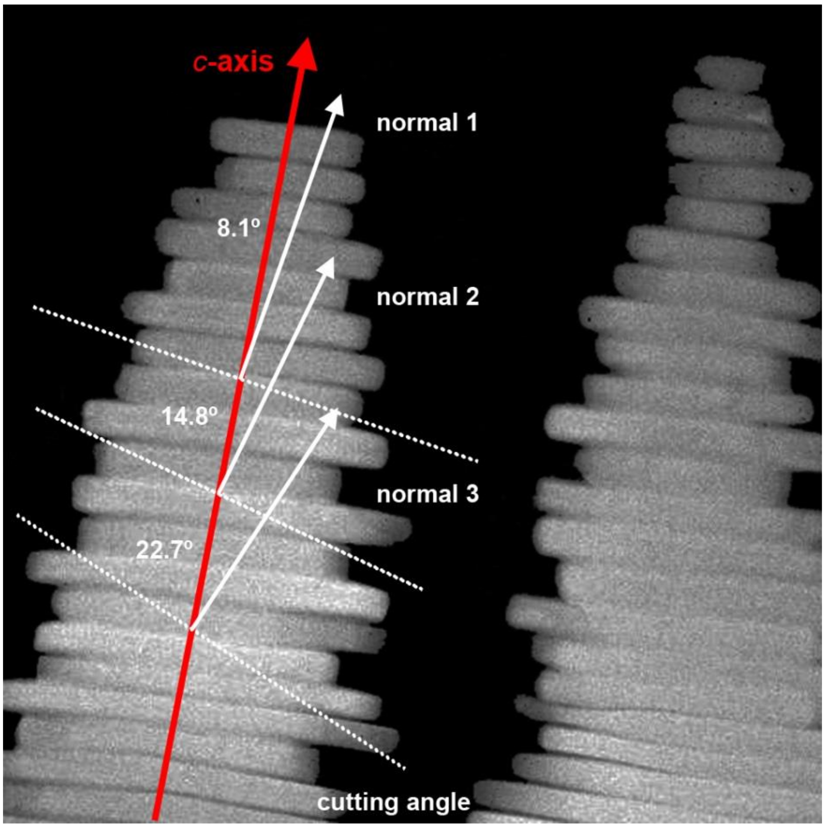


**Supplementary Fig. 7. Diagram indicating how the cutting angle affects the orientation of the particles.** When working without tilting the sample, the cutting angle largely determines the closest zone axis to index the particles. The normal to the cutting plane aligns in parallel to the optical axis of the microscope column, but does not necessarily coincide with a zone axis. Thus, the angle formed by the *c*-axis and the closest zone axis differs depending on the cutting angle. Only when the cut is exactly perpendicular to the nacre column growth axis, we are able to index the particles along the [001] zone axis. In our samples, the angles between the *c*-axis of the nacre column and the zone axis of the indexed particles varied from 0º to 24º (n = 12 slices). With a double tilt holder it is possible to tilt the sample so as to orient the particle along a particular zone axis, but this is a time-consuming procedure and therefore difficult to achieve in small crystalline domains without inducing beam damage.

**Supplementary Table 1.** Approximate transitions in carbon K-edge, calcium $L_{2,3}$ edge and oxygen K-edge fine structure and assignment of peaks. Peaks in bold are the most characteristic for each edge.

| Approximate Energy Loss (eV) | Peak assignment |
|---|---|
| **Carbon** | |
| 285.0 | C1s → π* in C=C |
| 287.5 | C1s → σ* in C–H |
| 288.4 | C1s → π* in C=O of carboxyl groups |
| **290.3** | C1s → π* transitions of carbon-oxygen bonds |
| 295.5 | C1s → π* transitions of carbon-oxygen bonds |
| 298.3 | C1s → π* transitions of carbon-oxygen bonds |
| **301.5** | C1s → σ* transitions of carbon-oxygen bonds |
| **Calcium** | |
| **349.3** | Ca $L_3$ edge |
| **352.6** | Ca $L_2$ edge |
| **Oxygen** | |
| **534** | O1s → π* transitions from C=O bonds |
| 540 | O1s → σ* transitions of the $CO_3^{-2}$ group |
| 541 | O1s → σ* transitions of the $CO_3^{-2}$ group |
| 544 | O1s → σ* transitions from the C=O |
| 545.5 | O1s → σ* transitions from the C=O |
